# Quadriceps Tendon Autograft Anterior Cruciate Ligament (ACL) Reconstruction With Internal Brace Augmentation: A Case Series

**DOI:** 10.7759/cureus.93030

**Published:** 2025-09-23

**Authors:** Miles C Farlow, Christopher C Rector, Michael McGraw

**Affiliations:** 1 Orthopaedic Surgery, East Carolina University Health, Greenville, USA; 2 Orthopaedic Surgery, Orthopedics East, Greenville, USA

**Keywords:** acl reconstruction, cutting athletes, graft selection, internal brace augmentation, quadriceps autograft, re-rupture rate, return to play, surgical outcomes

## Abstract

Introduction: Anterior cruciate ligament reconstruction (ACLR) is among the most common knee procedures in the United States, particularly in athletes who participate in cutting and pivoting sports. While bone-patellar tendon-bone (BTB) autografts have long been considered the gold standard for this group, drawbacks such as anterior knee pain and difficulty kneeling have prompted interest in quadriceps tendon autografts. With a larger cross-sectional area and higher load-to-failure, the quadriceps autograft is a promising alternative. This study reported return-to-play timelines and re-rupture rates in 44 cutting and pivoting athletes who underwent ACLR using quadriceps tendon autograft with internal brace augmentation (QAIB), offering early insight into its potential as an alternative to BTB autografts.

Methods: A retrospective chart review was performed on cutting and pivoting athletes who underwent ACLR using QAIB. Athletes were included if they participated in organized sports or sustained their injury while engaging in a cutting and pivoting sport. Five sports were classified as cutting and pivoting: football, soccer, basketball, lacrosse, and rugby. Patients were excluded if postoperative care was transferred to outside providers. Through chart review, time to return and re-rupture rates were collected. Time to return was defined by either direct documentation of the return date or the earliest documented clearance for return. Patients lacking this data were excluded from the time-to-return analysis. One patient was omitted due to a postoperative infection requiring graft removal and revision with a non-quadriceps autograft. Patients undergoing additional ligamentous reconstructions, such as posterolateral corner repairs, were also excluded from time-to-return calculations due to prolonged expected recovery.

Results: A total of 44 athletes were included, with an average age of 20.3 years. Of these, 29 (65.9%) were male and 15 (34.1%) were female. Eleven (25%) were Division I athletes. The most common sports were football (n=15) and basketball (n=15), followed by soccer (n=11), lacrosse (n=2), and rugby (n=1). Concomitant meniscal surgery was performed in 22 patients (50%). Time-to-return data was available for 36 athletes (81.8%). Seven lacked sufficient documentation, and one patient was excluded from the study entirely due to postoperative infection requiring graft revision. Two patients were excluded from the time-to-return analysis due to concurrent posterolateral corner reconstructions. The average time to return was 240.5 days (range: 166-312). Among Division I athletes (9/11, 81.8%), the average time to return was 255.0 days (range: 181-312), compared to 234.6 days (range: 166-309) for non-Division I athletes (27/33, 81.8%). No re-ruptures occurred. Four athletes (9.1%) developed arthrofibrosis requiring manipulation under anesthesia: one female athlete (basketball) and three male athletes (two football, one basketball)

Conclusion: QAIB ACLR in cutting and pivoting athletes demonstrated a 0% re-rupture rate and return-to-play timelines comparable to existing standards. While early results are promising, limitations in data capture, particularly return-to-play documentation and patient-reported outcomes, highlight the need for more robust, prospective studies to better assess long-term efficacy and safety.

## Introduction

Anterior cruciate ligament reconstruction (ACLR) is one of the most frequently performed orthopedic procedures in the United States, particularly among athletes. Over the past two decades, the incidence of anterior cruciate ligament (ACL) tears and subsequent reconstructions has continued to rise, especially in young and active populations. Between 2002 and 2014, the rate of ACLR increased significantly across all age groups, with a particularly steep rise observed in female adolescents [1,2]. National registry data show that a large proportion of individuals with ACL injuries eventually undergo surgical reconstruction, with incidence rates ranging from 32 to 68 per 100,000 person-years depending on demographic factors [3,4]. This growing burden underscores the importance of optimizing surgical techniques and graft selection to improve outcomes and reduce complications in high-demand populations.

ACLR techniques are especially important in high-level cutting athletes, as the incidence of ACL tears in this population is higher than in the general population and has continued to rise [5]. Bone-patellar tendon-bone (BTB) autografts have historically been favored in this group due to their high fixation strength and favorable long-term stability. However, BTB grafts are associated with increased rates of anterior knee pain, kneeling discomfort, and potential for patellar fractures or tendon rupture, prompting interest in alternative graft options [6]. Hamstring tendon autografts offer reduced anterior knee symptoms but may be prone to tunnel widening, decreased fixation strength, and lingering hamstring weakness in active patients. Over the past decade, quadriceps tendon autografts have gained popularity, particularly in younger and athletic patients, due to their robust biomechanical properties, including a larger cross-sectional area and higher load-to-failure compared to BTB and hamstring grafts [7]. Additionally, quadriceps grafts offer lower donor-site morbidity and comparable clinical outcomes in terms of graft survival, knee stability, and return-to-play (RTP) metrics [8]. Recent comparative studies suggest that quadriceps tendon grafts may offer a more favorable balance of low complication rates and high patient satisfaction scores, with reduced anterior knee pain compared to BTB grafts and fewer strength deficits than hamstring autografts [9]. 

Internal brace augmentation has emerged as a promising adjunct in ACLR, particularly in athletic populations where minimizing graft failure and expediting return to sport are critical. The technique involves reinforcing the reconstruction with high-strength braided suture tape secured with knotless bone anchors, acting as a secondary stabilizer during the early healing phase. This construct provides load-sharing support, improves graft construct stiffness, and reduces elongation under cyclic loading, thereby minimizing the risk of early graft failure. Unlike traditional methods requiring graft harvest, internal bracing preserves native tissue and permits early mobilization without the need for external bracing. Systematic reviews have reported favorable clinical outcomes, demonstrating that internal brace augmentation is associated with low re-injury rates and high rates of patient satisfaction, particularly when combined with ligament repair or reconstruction [10,11]. Early clinical data from patients undergoing combined ACL and anterolateral ligament repair with internal brace support show significant improvements in joint stability and patient-reported outcomes at a minimum two-year follow-up [12]. These findings suggest that internal bracing may enhance graft protection during rehabilitation and allow for more confident progression through sport-specific activity. 

Despite the growing use of quadriceps tendon autografts and the emergence of internal brace augmentation as a supportive technique, few studies have examined outcomes in high-demand athletic populations using this combined approach. This study aims to evaluate RTP timelines and re-rupture rates in 44 athletes participating in cutting and pivoting sports who underwent ACLR with quadriceps autograft and internal brace augmentation (QAIB). Cutting and pivoting sport athletes were chosen due to the theoretical increased stress on ACLR grafts that this population may have. By focusing on this specific patient population, the study provides early insight into the potential of QAIB as a viable alternative to BTB autografts, one that may preserve biomechanical strength while reducing donor-site morbidity. Furthermore, it explores the added benefits internal bracing may offer in terms of graft protection and rehabilitation confidence. These findings may help guide surgical decision-making and contribute to a growing body of evidence supporting tailored graft and augmentation strategies in athletic ACLR. Given the novelty of this combined technique and the limited existing literature, a retrospective case series design was appropriate to generate preliminary data on outcomes in this emerging area.

## Materials and methods

A retrospective chart review was conducted at East Carolina Health Hospital, Greenville, North Carolina, to evaluate outcomes in athletes who underwent ACLR using QAIB between January 1, 2019, and October 3, 2023, at a single orthopedic practice. The study was approved by the University & Medical Center Institutional Review Board, East Carolina University (reference number: UMCIRB 23-001945), and informed consent was waived due to the retrospective nature of the review.

Patients were eligible for inclusion if they sustained an ACL tear while participating in a cutting or pivoting sport or had documented involvement in such activities at the time of injury. Cutting or pivoting sports involve frequent changes of direction. Five sports were defined as cutting and pivoting: football, soccer, basketball, lacrosse, and rugby. Athletes were included only if complete operative documentation confirmed the use of a QAIB. Exclusion criteria included patients who transferred postoperative care outside the institution, those with insufficient RTP data, and those who underwent additional ligamentous reconstructions that might confound recovery time, such as posterolateral corner repairs.

All ACLRs were performed using a standardized surgical technique involving QAIB using SwiveLock® anchors (Arthrex, Inc., Naples, Florida, United States). Graft preparation, tensioning, and tunnel placement were performed according to current best practices and remained consistent across all cases.

Chart review was conducted through March 1, 2025, which represented the last date of available access to the practice’s electronic health record. Follow-up duration for each patient was calculated from the operative date to this cutoff. Demographic data, including age, sex, sport, and Division I status, were collected. Operative details such as concurrent meniscal procedures were also recorded. The primary outcome measures were RTP timing and re-rupture rate.

RTP was defined by either direct documentation of the RTP date in the clinical chart or the earliest documented clearance or recommendation to return to sport. The participating surgeons used individualized determinations based on a combination of standardized clinical guidelines, functional performance testing, and patient-reported readiness. However, due to inconsistent documentation and limited availability of performance testing data across patients, it was not possible to uniformly extract detailed RTP criteria for each case. As such, RTP dates were identified using the most reliably documented evidence of clearance. Patients without clear documentation of RTP were excluded from the time-to-return analysis. We did not perform routine telephone follow-up or systematically obtain outside records; therefore, reruptures managed at other institutions may be under-ascertained. One patient who developed a postoperative infection requiring debridement and revision ACLR with a non-quadriceps graft was excluded entirely from analysis. Two additional patients were excluded from RTP analysis due to concurrent posterolateral corner reconstructions. Group differences (included vs excluded) were assessed using Welch’s t-test for age and Fisher’s exact tests for categorical variables.

Secondary outcomes included complications such as arthrofibrosis requiring manipulation under anesthesia and concurrent meniscal surgery. All outcomes were assessed through electronic medical record (EMR) review. Descriptive statistics were used to report demographic variables, surgical details, and outcome measures. Continuous variables such as age and time to RTP were reported as means with ranges. Categorical variables, including sex, sport type, and complication rates, were reported as counts and percentages. Subgroup analysis was performed comparing RTP times between Division I and non-Division I athletes. Due to the descriptive nature of this case series, no hypothesis testing or inferential statistics were applied.

## Results

A total of 44 athletes met the inclusion criteria for this study. Of these, 29 (66%) were male and 15 (34%) were female. The average age of participants at the time of surgery was 20.3 years (range: 15-30). Eleven athletes (25%) were identified as Division I participants based on documentation of collegiate sport status. The most common sports represented were football (n=15) and basketball (n=15), followed by soccer (n=11), lacrosse (n=2), and rugby (n=1). Concomitant meniscal surgery was performed in 22 athletes (50%) at the time of ACLR (Table 1). 

**Table 1:** Patient demographics and sports distribution

Variable	Values
Total athletes	44
Age at Surgery (years), mean (range)	20.3 (15-30)
Sex (Male/Female), n (%)	29 (66%)/15 (34%)
Division 1 Athletes, n (%)	11 (25%)
Sports, n (%)	
Football	15 (34%)
Basketball	15 (34%)
Soccer	11 (25%)
Lacrose	2 (4.5%)
Rugby	1 (2.7%)
Concomitant Meniscal Surgery, n (%)	22 (50%)

**Figure 1:**
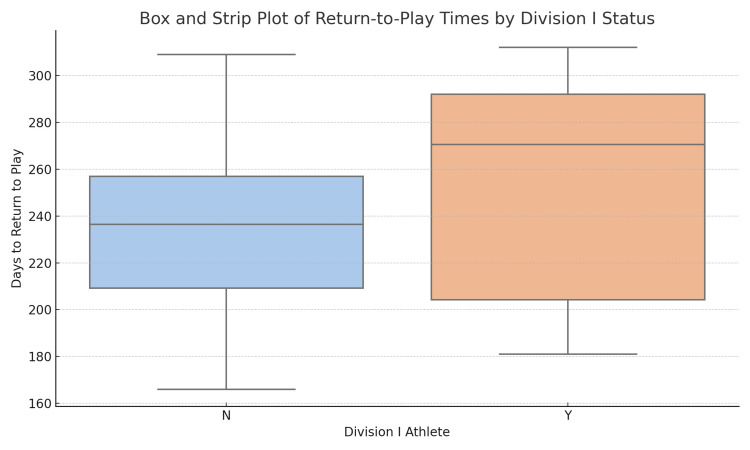
Box and strip plot illustrating return-to-play times (in days) stratified by Division I status. Each point represents an individual athlete. The box represents the interquartile range and the median.

Time-to-return data was available for 36 out of 44 athletes (81.8%). Among athletes with available RTP data (n=36), the overall mean time to RTP was 239.7 ± 41.0 days (95%CI, 225.8-253.5; range, 166-312). Division I athletes (n=9) returned at an average of 255.0 ± 48.1 days (95%CI, 218.0-292.0; range, 181-312), while non-Division I athletes (n=27) returned at 234.6 ± 38.0 days (95%CI, 219.5-249.6; range, 166-309) (Table 2). Figure 1 illustrates this distribution of RTP times, showing that Division I athletes tended to return later and exhibited a slightly wider distribution compared to their non-Division I counterparts.

**Table 2:** Outcomes and return to play

Variable	Value
Time to return (all), mean (range)	240.5 days (166–312)
Time to return (D1 athletes), mean (range)	255.0 days (181–312)
Time to return (non-D1 athletes), mean (range)	234.6 days (166–309)
Re-rupture rate	0%
Arthrofibrosis requiring MUA, n (%)	4/44 (9.1%)

At a mean follow-up of 2.8 ± 0.9 years (range, 1.4-5.4), no re-ruptures were observed (0/44; 0.0%; 95% CI, 0.0-8.0). Arthrofibrosis requiring manipulation under anesthesia occurred in 4/44 (9.1%) (95%CI, 2.5-21.7). These included one female basketball player and three male athletes (two football, one basketball). No other complications leading to graft failure or revision were identified in the remaining sample.

Seven patients were excluded due to incomplete RTP documentation, and one additional patient was excluded from all analyses due to a postoperative infection requiring graft removal and revision with a non-quadriceps autograft. Two patients who underwent concurrent posterolateral corner reconstructions were excluded from return-to-play calculations due to the potential for extended recovery timelines.

Athletes excluded from the RTP analysis were older than those included (mean 20.6 vs 17.8 years; mean difference Δ = 2.8 years, 95%CI 0.2-5.4; p = 0.035). In contrast, no other baseline characteristics, including sex, Division I status, concomitant meniscal surgery, laterality, or ALLR augmentation, differed significantly between included and excluded athletes. Sport-specific percentages for exclusion are presented descriptively and should not be over-interpreted (Table 3).

**Table 3:** Baseline characteristics of athletes included vs excluded from RTP analysis Notes: Data are presented as mean ± SD or n (%). P-values: Welch’s t-test for age; Fisher’s exact test for binary variables

Characteristic	Included (n=36)	Excluded (n=8)	p-value
Age (years), mean ± SD	17.8 ± 3.0	20.6 ± 3.0	0.035
Male, n (%)	23 (63.9%)	6 (75.0%)	0.695
Division I, n (%)	9 (25.0%)	2 (25.0%)	1.000
Concomitant meniscal surgery, n (%)	19 (52.8%)	3 (37.5%)	0.698
ALLR/ALL augmentation, n (%)	10 (27.8%)	1 (12.5%)	0.656
Sport, n (%)			
Basketball	12 (33.3%)	3 (37.5%)	
Football	13 (36.1%)	2 (25%)	
Lacrosse	2 (5.5%)	0 (0%)	
Rugby	1 (2.7%)	0 (0%)	
Soccer	8 (22.2%)	3 (37.5%)	

## Discussion

This study demonstrates favorable early outcomes for ACLR using QAIB in a cohort of cutting and pivoting athletes. Notably, the cohort experienced a 0% re-rupture rate, and average RTP timelines were comparable to previously published data for both BTB and quadriceps autografts. These findings suggest that QAIB may be a viable and durable graft option in high-demand athletic populations, particularly when minimizing donor-site morbidity and graft failure are clinical priorities.

Quadriceps tendon autograft has gained increasing attention in recent years due to its larger cross-sectional area, higher load-to-failure, and reduced anterior knee pain compared to BTB autografts [7,8]. This is especially relevant in cutting and pivoting athletes, where graft choice must balance biomechanical performance with RTP expectations. Compared to BTB and hamstring autografts, quadriceps tendon grafts have demonstrated similar or superior functional outcomes with significantly lower rates of anterior knee pain and donor-site complications, reinforcing their utility in active patient populations [9]. The average time to return in our series was 240.5 days, which aligns well with existing timelines reported in the literature for both BTB and quadriceps grafts in similar athletic populations [6-8]. 

Division I athletes in our cohort had a slightly longer average RTP time (255.0 days) compared to non-Division I athletes (234.6 days), a trend that may reflect institutional clearance policies, greater caution in rehabilitation, or higher physical demands prior to return. This difference, while not statistically analyzed due to the descriptive nature of the study, is consistent with previously observed patterns in elite athletes and reinforces the importance of individualized recovery protocols [7]. Although confidence intervals were provided to illustrate the precision of our estimates, the relatively small sample size, particularly within subgroups, and low event counts mean these intervals should be interpreted with caution. Figure 1 illustrates this trend visually, demonstrating broader variability and a slightly right-shifted distribution in Division I participants.

The use of internal brace augmentation may have contributed to the favorable outcomes observed in this series. Biomechanical data have shown that internal bracing reduces graft elongation and enhances construct stability during the early phases of healing [10,11]. Clinical studies have reported low re-injury rates and improved confidence during rehabilitation when internal bracing is used as an adjunct to ACLR [11,12]. In this study, no graft re-ruptures occurred despite a high percentage of patients returning to high-risk sports, suggesting potential protective benefits of the internal brace during early graft incorporation and load bearing.

The incidence of postoperative arthrofibrosis requiring manipulation under anesthesia was 9.1%, which is within the reported range in the ACL literature [10-12]. While not directly attributable to the graft or internal brace, this finding highlights the importance of closely monitored rehabilitation to balance protection and mobility in the early postoperative period.

This study has several limitations. As a retrospective, single-institution case series, it lacks a control group and is subject to selection and documentation biases. Return-to-play dates were determined by chart review and, in some cases, based on clearance recommendations rather than confirmed athletic participation. Additionally, patient-reported outcomes and objective strength testing were not available for analysis, limiting the ability to draw conclusions about functional recovery. Also, although no re-ruptures were identified, the lack of a standardized follow-up period means late failures may not have been captured. There was a statistically significant age imbalance between included and excluded athletes. Because exclusion was due to missing RTP documentation, this could introduce selection bias if age is associated with RTP timing or documentation quality. One plausible explanation is that older patients are less engaged in organized, clearance-dependent sport, leading to fewer formal clearances and, consequently, fewer EMR-documented RTP dates; this mechanism cannot be verified with our available data. Furthermore, although all procedures were performed by board-certified orthopedic surgeons, variability in surgical technique may exist, particularly given the learning curve associated with quadriceps tendon harvest and internal brace fixation. There was also no consistent documentation between surgeons that included graft diameter, exact internal brace configuration, or other technical aspects of the surgery. These technical factors could influence outcomes and should be considered when interpreting the results. 

Despite these limitations, this case series contributes early evidence supporting QAIB as a promising option in athletic ACLR. The absence of re-ruptures and acceptable RTP timelines suggests that this technique warrants further prospective evaluation in larger cohorts and randomized comparisons to traditional grafts and fixation strategies. These findings may also hold clinical relevance for graft selection in high-demand athletic populations, such as collegiate or high school athletes engaged in cutting and pivoting sports. QAIB offers a compelling balance of biomechanical strength, reduced donor-site morbidity, and potential early-phase graft protection through internal bracing. In this context, QAIB could represent a viable alternative for athletes for whom graft durability, accelerated rehabilitation, and minimized complication risk are critical. As such, this approach may warrant future consideration in ACLR treatment algorithms tailored to high-risk athletic populations.

## Conclusions

In this retrospective chart review of cutting and pivoting athletes, ACLR using QAIB resulted in a 0% re-rupture rate and RTP timelines consistent with those reported for traditional graft techniques. These findings support QAIB as a promising alternative to BTB autografts, particularly in athletes where donor-site morbidity and graft durability are critical considerations.

While the results are encouraging, limitations in retrospective data capture and the absence of long-term functional outcomes underscore the need for further prospective study. Future research should evaluate the durability of QAIB in larger cohorts and explore patient-reported and performance-based outcomes over time. Internal bracing may offer added value in enhancing early graft protection, and its role in modern ACLR warrants continued investigation.
